# Avian Mycobacteriosis: Still Existing Threat to Humans

**DOI:** 10.1155/2016/4387461

**Published:** 2016-07-31

**Authors:** Michal Slany, Vit Ulmann, Iva Slana

**Affiliations:** ^1^Veterinary Research Institute, Hudcova 70, 621 00 Brno, Czech Republic; ^2^Institute of Public Health in Ostrava, Partyzanske Namesti 7, 702 00 Ostrava, Czech Republic

## Abstract

The nontuberculous mycobacteria are typically environmental organisms residing in soil and water. These microorganisms can cause a wide range of clinical diseases; pulmonary disease is most frequent, followed by lymphadenitis in children, skin and soft tissue disease, and rare extra pulmonary or disseminated infections.* Mycobacterium avium* complex is the second most common cause of pulmonary mycobacterioses after* M. tuberculosis*. This review covers the clinical and laboratory diagnosis of infection caused by the members of this complex and particularities for the treatment of different disease types and patient populations.

## 1. Introduction

Mycobacteria are acid-fast Gram-positive bacilli characterized as intracellular parasites. As of today, 169 mycobacterial species and 13 subspecies have been identified [1]. This large group includes obligate pathogenic mycobacteria causing tuberculosis (members of the* Mycobacterium tuberculosis* complex; MTC) and leprosy (*M. leprae*), as well as nontuberculous mycobacteria (NTM), occurring widely in the environment [2].

The Czech Republic has been certified to be free from bovine tuberculosis since 2004 [3]. It should be noted that tuberculous lesions have still been found in the lymph nodes and sporadically in the parenchymal organs of livestock (mainly cattle and pigs). When mycobacterial isolates from these tuberculous lesions were subjected to analysis, it was found that more than 95% were members of the* Mycobacterium avium* complex (MAC, [4]).

MAC are isolated increasingly in immunocompetent individuals [5]. Possible explanations include an increase in the number of individuals with lung damage making them more susceptible to mycobacterial infection, environmental exposure, or improved laboratory diagnostics.

An extensive analysis of the current literature has shown that members of the MAC are widely spread. In particular, two mycobacterial species belonging to the MAC (*M. avium* subsp.* hominissuis* and* M. intracellulare*) can be successfully propagated in the environment in high concentrations under certain conditions [6]. It should be, however, noted that even other nontuberculous mycobacteria can be potentially pathogenic (PPM) for humans and animals [4].

The natural reservoirs of these primarily nonpathogenic mycobacteria are aquatic and terrestrial environments [2]. Most of the PPM are able to produce biofilm, which is probably the main factor influencing their long-term survival in the environment and the emergence of possible sources of infection for humans and animals [7].

Infections caused by PPM are on the increase because of changes in lifestyle, environment, and other factors which affect the susceptibility of humans to disease. Infection with PPM is mainly manifested in functionally predisposed and immunocompromised individuals [8]. Most often it is a secondary immunodeficiency due to HIV infection or immunosuppressive treatment after transplantation or other systemic diseases [9]. The host is usually infected via inhalation of contaminated aerosols or via contact through subcutaneous skin abrasions [8]. PPM virulence is lower compared to* M. tuberculosis* [10], but in some cases, infection may have an aggressive course with systemic dissemination [11]. Interhuman transmission of PPM has not been demonstrated yet [6].

The aim of this review article is to explain the MAC taxonomy and summarize the occurrence of infections caused by MAC members in humans and animals.

## 2. Taxonomy of* M. avium* and Other Mycobacterial Species Belonging to MAC

In the past, the classification of mycobacteria was based upon different morphological and phenotypic characteristics of individual mycobacterial species. The classic approach uses the scheme according to Runyon that differentiates mycobacterial species on the basis of colony growth rate and ability to form pigments of different colors [15]. In the last two decades, there has been a huge expansion of molecular-based methods, which has facilitated the discovery of new mycobacterial species and has led to changes in taxonomic classification [6]. Two major taxonomic groups of clinical significance are MTC and MAC (Table 1).

Seven out of the eight members of the first group (MTC) can cause disease in humans. In the Czech Republic* M. tuberculosis*,* M. bovis*,* M. caprae,* and* M. pinnipedii* have been isolated from humans and from animals [16, 17]. The incidence of human tuberculosis shows a decreasing trend in the Czech Republic, but there are new threats in the form of multidrug resistant forms of the disease. Human tuberculosis caused by* M. bovis* was no longer important after the eradication of bovine tuberculosis in cattle in 1968, although sporadic cases in humans still occur in the Czech Republic [17].

In the past, serotyping was widely used to characterize members of the MAC. A total of 28 serotypes have been described [18]. MAC species have lower pathogenicity in humans compared to the members of the MTC. These species are primarily found in the environment and in birds. The MAC comprises a number of slow-growing environmental and animal-associated mycobacterial species responsible for opportunistic human infections. The three main members are

- *M. avium *subsp.* avium *(serotypes 1–3 of former* M. avium*),
- *M. avium* subsp.* hominissuis *(serotypes 4–6, 8–11, and 21 of the former* M. intracellulare*),
- *M. intracellulare* (the remaining serotypes 7, 12–20, and 22–28 of MAC).

All these species are responsible for infections especially in birds and pigs [19].* Mycobacterium avium* subsp.* hominissuis* (MAH) is the most commonly detected NTM causing infection in humans and pigs [19].* Mycobacterium avium* subsp.* avium* (MAA) occasionally infects pigs and humans but is generally regarded as an obligate pathogen of birds, causing contagious avian tuberculosis [20].* Mycobacterium intracellulare* (MI) is an environmental organism associated with lymphadenopathy (enlarged lymph nodes) in children and progressive pulmonary disease in elderly women [21]. MAC currently comprises seven additional species.* M. chimaera* (formerly sequevar MAC-A) and* M. colombiense* (formerly sequevar MAC-X) were first isolated from clinical samples from patients in Italy and Colombia [22, 23].* M. vulneris* was firstly described as a new species isolated from wound infection in elderly woman and from cervical lymphadenitis in two-year-old girl [24].* M. arosiense* was recently uncovered as a cause of pulmonary infection and osteomyelitis [25, 26]. First two cases of pulmonary infection associated with* M. marseillense* were reported two years later [27, 28]. Nowadays, only one case describing infection of* M. timonense* in an HIV positive man was reported [29]. Clinical relevance of* M. bouchedurhonense* is still unknown due to the clinically related but not confirmed isolate [30].

## 3. Patterns of Human Infection Caused by* M. avium* Complex Agents

The incidence of avian mycobacterioses in humans has shown an increasing tendency as human and bovine tuberculosis has begun to be eradicated in developed countries. This trend was also certainly affected by the development of diagnostic methods. It is possible to distinguish four forms of disease caused by mycobacteria belonging to the MAC.

### 3.1. Lung Disease

Lung disease is the most common form of infection caused by members of the MAC.

The most common and well-recognized pattern is cavitary disease, predominantly involving the upper lobes, similar to the pulmonary TB seen in sanitarium patients [8, 31]. Patients with MAC lung disease tend to be older than TB patients and are not infectious for other humans [32]. They are predominantly male, often with a history of tobacco and/or alcohol abuse, and usually have underlying lung disease [33]. First large comprehensive study describing main patterns was published in 1979. Authors reported that 12% of patients with MAC demonstrated a reticular, fibrotic radiograph rather than cavitary disease. In contrast to the patients with cavitary disease, patients with fibronodular bronchiectasis are predominantly elderly women, often without a history of smoking or underlying lung disease [34]. Rare condition that can occur in young to middle aged patients is a solitary nodular lesion resembling tuberculoma or malignancy, indistinguishable by imaging [35]. Pleurisy caused by MAC is an uncommon recently recovered state [36, 37]; in that case, the standard microbiological methods are decisive for an adequate treatment.

#### 3.1.1. Hypersensitivity Pneumonitis-Like Illness (Hot Tub Lung)

Many cases of other forms of lung disease resembling hypersensitivity pneumonitis (HP) started to be reported at the end of the last century [38, 39]. The common denominator of these cases is the use of rehabilitation swimming pools and hot tubs before the onset of clinical symptoms. Simultaneously, MAC species have been isolated from clinical samples and from water environments. The pathogenesis of this so-called “hot tub lung” (HTL) is still disputed. Some authors indicate HTL as “hypersensitivity pneumonitis-like disease” [40]. The main emphasis is focused on the theory of hypersensitivity rather than on the infection caused by live MAC cells. Clinically, the disease first appears as flu-like symptoms and gradually progresses to cough, shortness of breath, fever, and night sweats. The symptoms become worse after exposure to aerosols in the hot tub. Some patients experience healing after they avoid contaminated hot tubs or after corticosteroid remedy [38].

As pointed out by Agarwal and Nath, histopathological changes in the lungs of HP patients are different from changes presented by HTL patients [39]. The development of HP is accompanied by a histological triad (cellular bronchiolitis, chronic interstitial pneumonitis, and isolated small granulomas). Radiography of the chest usually discloses dominant interstitial inflammation with poorly defined granulomas. In contrast, HTL patients present strong and well-defined granulomas overshadowing interstitial inflammation.

The hypersensitivity reaction is probably triggered by antigens from both the living and dead MAC cells that enter the respiratory route through aerosols produced by air pumping in the hot tub. Similarly, other forms of pulmonary hypersensitivity are caused by inhalation of bacterial or fungal antigens or antigens from the environment: “humidifier lung,” “farmer's lung,” or “lung of pigeon breeders” [41].

### 3.2. Soft Tissue and Skin Lesions

Skin lesions are a rare form of disease caused by MAC. They occur particularly in patients with primary disease, which weakens their immune system (e.g., diabetes mellitus, cancer, chronic renal failure, HIV/AIDS, and Immune Reconstitution Inflammatory Syndrome (IRIS)). Infection results in plaques, nodular changes, and subcutaneous abscesses or can even resemble skin changes caused by leprosy [42, 43]. A spindle cell pseudotumor of the skin after infection by* M. intracellulare* was also reported [44].

### 3.3. Peripheral Lymphadenopathy

Another relatively common form of disease caused by MAC is peripheral lymphadenopathy, which mainly affects children [45]. Lymphonodal infection is typically an infantile disease affecting, in most cases unilaterally, cervical lymph nodes [46]. The route of infection is most likely oral, and the childhood habit of bringing hands and objects to the mouth may well explain the particular susceptibility of infants to this pathology [47]. The size of neck swelling, which is painless, varies considerably and fistulization is not rare.

Interestingly, BCG vaccination may provide protection against infections caused by members of the MAC. For example, discontinuation of a BCG vaccination program resulted in an increase in nontuberculous mycobacterial infections in the Czech Republic and Sweden [48–50]. Recent observations lend further strong support to this idea. Calmetization in the Czech Republic was finally stopped in 2010, before any case of MAC cervical lymphadenitis had been observed. From June 2014 to today, five cases of 3-4-year-old children (two girls and three boys) have been confirmed, only in the Moravian region (unpublished data).

In 2009, the first case of cervical lymphadenopathy in a young girl caused by* M. colombiense* was described. Previously, this species was only linked with cases of respiratory disease and disseminated infections [51].

### 3.4. Disseminated Infections

Disseminated MAC infections develop predominantly in severely immunocompromised people; the best known are those that affect HIV-infected patients [52]. The use of highly active antiretroviral therapy has reduced the incidence of opportunistic infections, including MAC [53]. However, the health status of some patients paradoxically deteriorates due to the IRIS. The restoration of immune function together with an augmentation of the levels of CD4 + T-cells in the blood could play a negative role, because the opportunistic infection, which is already present, may affect the formation of inflammatory responses, thus leading to disease. MAC members very often play a major role in this syndrome [54].

## 4. Risk Factors for MAC Infection in Humans

Adequate management of patient's case requires evaluating mycobacterial isolate together with further clinical circumstances (Table 2). Development of real infection relies on a number of predisposing conditions (e.g., altered immunity status, infection dose).

### 4.1. Primary Disease

Alterations of cell immunity mechanisms can be divided to primal and acquired. Inherited (Mendelian) dysfunctions, primarily most serious, are based on inadequate cytokine (IL-12, INF-*γ*) production and pathway or receptor abnormalities. It could lead to disseminated disease or relapses after another exposition when manifested in early age [55]. Diabetes mellitus predisposes patients in a way of altered migration of phagocytes and low cytokine production/response [56].

Acquired alteration of cell immunity mechanisms arises from different conditions characterized by an impaired immune system: HIV/AIDS, postoperative period, therapy with TNF-*α* antagonists, and corticotherapy [57].

### 4.2. Secondary Disease

Functional weakness, lung tissue alteration, skeletal malformation of chest, and ciliary disorders lead to deterioration of self-cleaning function and physiological barriers, which are fundamental for effective elimination of microorganisms from lower respiratory tract. Airways obstruction, pathological changes of lung parenchyma such as fibrosis or necrosis by silicosis, smokers chronic obstructive pulmonary disease [58], cystic fibrosis [59], and niche created during former tuberculosis allows to microbial accumulation, surviving, and growth. Alteration of tissue, mucus, and inhaled pollution could negatively influence defense abilities of alveolar macrophages [60]. Such conditions allow mycobacteria to colonize respiratory tract on surface deposits in alveoli without interaction for long time. Microbial overgrowth and immunity response may lead to exacerbation of undelaying condition or true illness [60, 61].

True mycobacteriosis, contrary to tuberculosis, is result of comorbidities and coincidences.

## 5. Treatment of MAC Infections

Generally, prophylaxis, medical, and surgical treatment are three important aspects of management of infections due to NTM (Table 3, [62]). MAC is intrinsically resistant to many antibiotics and antituberculosis drugs. Due to the structure of cell wall and membrane impermeability, many traditional and well-tolerated antimicrobial drugs (e.g., beta lactams) are not effective against MAC. Isoniazid as classical representative of antituberculotics has for the same reason a little importance in proper treatment [63]. Active efflux and heterogeneous mutations in genome result in primary or inducible resistance to curative drugs as macrolides, rifamycins, or ethambutol [64, 65]. To completely eradicate a mycobacterial infection with antibiotics requires that the course be long enough (at least 12 months) to act against bacteria that are dormant as well as active. Initial treatment of MAC disease should consist of two or more antimycobacterial drugs to prevent or delay the emergence of resistance [66]. Commonly used first-line drugs include macrolides (clarithromycin or azithromycin), ethambutol, and rifamycins (rifampin, rifabutin). Aminoglycosides, such as streptomycin and amikacin, are also used as additional agents [67].

### 5.1. Pulmonary MAC Infection

Guidelines stated by the American Thoracic Society recommend that most patients with pulmonary infection can be treated with a thrice-weekly triple drug regimen of clarithromycin (1000 mg) or azithromycin (500 mg), rifampin (600 mg), and ethambutol (25 mg/kg). Therapy should be continued for at least one year after culture results revert to negative [8]. In addition, the guidelines suggest the addition of amikacin or streptomycin thrice weekly early in the course of treatment (initial 2-3 months) in patients with severe lung disease. Fluoroquinolones should be used as second-line agents against MAC because of the poor outcome associated with them compared with macrolide-containing regimens [8].

### 5.2. Soft Tissue and Disseminated MAC Infection

The most active regimen according to the current guidelines seems to be a combination of clarithromycin (500 mg twice daily) and ethambutol (15 mg/kg daily) with or without rifabutin (300 mg daily). Azithromycin (500–600 mg daily) can be substituted for clarithromycin [8]. The addition of rifabutin has been recommended, especially in patients with advanced immunosuppression and high mycobacterial loads in blood or in the absence of effective antiretroviral therapy. Based on experience in patients without HIV infection, the guidelines suggest the use of amikacin or streptomycin as third or fourth drugs in these patients [68].

### 5.3. MAC Lymphadenitis

Antimicrobial treatment has a generally poor outcome, and a successful healing process in most cases may be supported by surgical excision of the lymph nodes involved [47]. The intervention, however, resolves the situation in 80% of cases, with the remaining 20% being characterized by relapse. Antibiotics are generally not required but may be beneficial in patients with extensive lymphadenitis or with a poor response to surgical therapy [69].

### 5.4. Chemoprophylaxis

Because 40% of patients with advanced HIV disease are likely to develop disseminated MAC, it makes sense to develop a strategy for preventing this disease in patients at risk [62]. The drug of choice is either clarithromycin 1000 mg per day or azithromycin 1200 mg per week [66].

### 5.5. Resistance to Macrolides

Administration of other effective drugs must necessarily be based on a sensitivity testing. In cases of rifamycins or macrolide resistance, clofazimine and quinolones instead or in combination with other sensitive drugs have proven good outcome [70].

## 6. Infection of Animals Caused by Members of the* M. avium* Complex

MAC members are widely distributed in the environment and have been isolated from soil, wastewater, water tanks, aerosols, protozoa, vegetation, animals, and humans [68]. Mycobacteria belonging to the MAC can affect a wide range of wild animals, but little has been published on the clinical signs, which are rarely perceived or not documented.

MAA is one of the causative agents of avian mycobacteriosis in birds. Domestic poultry are highly susceptible to MAA and are often the first to contract the disease, acting as a primary reservoir for the bacterium [71, 72]. Other than birds, MAA infection has been reported in various other mammalian species, including small rodents, dogs, cats, pigs, cattle, and horses [73–76]. Mammals are, however, affected only sporadically [73]. Direct transmission between infected animals supports the classification of MAA as a zoonotic agent [71].

In contrast, sources of MAH and MI are mainly various components of the environment, which are under certain circumstances massively contaminated by these agents [77]. Transfer of these two mycobacterial species between infected animals has been reported sporadically [6]. MAH has also been isolated from lesions in deer and MI was also found in deer species although not as commonly and infection is usually subclinical [78].

The spread of MAC-caused disease in herds of livestock poses a big threat to the breed. Considerable spread of the causative agent not only to animals but also in the environment could occur during the long incubation period of the disease. Under such circumstances, the disease agent can become a source of infection for newborns and also for other animals of various species [79]. The clinical significance of individual MAC members varies between livestock species. Domestic poultry is highly susceptible to infection with* M. avium*, the causative agent of avian tuberculosis [80]. The disease is occasionally observed in the small-scale backyard breeding of poultry [71]. In commercial poultry farms, the disease has been eradicated due to the rapid turnover of flocks and good hygienic conditions. In general, three forms of disease caused by* M. avium* are observed in birds:

- granulomatous lesions in various organs,
- “paratuberculosis” form with lesions in the intestinal tract, accompanied by excretion of high doses of microorganisms,
- atypical form, which is usually observed in finches, canaries, and small parrots.

Regarding infection of MAC in pigs, MAH is the absolutely most isolated pathogen, while MAA is not so often detected. MAH infection in pigs usually has no clinical signs but can pose economic problems. MAH causes pathological changes most commonly in the mesenteric, head, or neck lymph nodes. Such affected parenchymal organs or sometimes the whole body of the animal is then confiscated by the veterinary authority. The source of infection for pigs is mainly an external environment (bedding, feed, feed water, soil, etc.) contaminated by droppings of wild birds or small terrestrial mammals [77, 81].

Horses are relatively resistant to MAC infections. Documented cases of MAA infection in horses have shown mainly granulomatous inflammation of various lymph nodes, intestine, and parenchymatous organs or tuberculous changes in the vertebrae [82]. In contrast, MAH infection in horses usually results in diffuse granulomatous enterocolitis, infliction of lung and lymph nodes, or even disseminated tuberculous changes in many organ systems, including the eye and heart [83].

Tuberculous lesions caused by* M. avium* in cattle are indistinguishable from lesions induced by* M. bovis*, which could complicate the diagnosis of new cases of* M. bovis* in countries free of bovine tuberculosis [84]. Granulomatous lesions are mainly localized in the lymph nodes of the digestive and respiratory systems [85], although cases of generalized disease have also been documented [74]. Ruminants are not infected by MAC members as frequently as by MAP, whose role in the pathogenesis of Crohn's disease in humans is still under discussion [86]. MAP is the causal agent of a chronic inflammation of the intestine in ruminants termed paratuberculosis (Johne's disease). In infected animals, it causes emaciation accompanied by diarrhoea, which can end with the death of the animal. The economic losses in infected cattle herds, especially in dairy herds, are enormous [87].

Hobby breed animals as dogs and cats are rarely infected by members of the MAC. The development of the disease is mainly associated with immunocompromised animals. MAC infections in cats were usually associated with both cutaneous lesions and generalized disease [88, 89].

Dogs possess a relatively high natural resistance to infection with MAC. However, a few sporadic occurrences of* M. avium* infections in dogs in the form of pulmonary, gastrointestinal, cutaneous, and disseminated lesions have been reported [90].

Today, red deer are often held in differently sized herds on pastures. The lack of a forest area stresses the animals, leading to an increased sensitivity to various diseases, including infections caused by* M. avium* [91].

## 7. Sources of MAC for Humans

Surface and drinking waters are naturally inhabited by multiple mycobacterial species and thus this environment could form an important part of the transmission route leading to MAC infection in humans [48]. MAH and MI are especially abundant in natural habitats, such as biofilms and sediments in rivers, ponds, and reservoirs with drinking water [45, 69]. Moreover, there are many reports on the isolation of potentially pathogenic mycobacteria (including MAC) from water systems in households and hospitals [92]. Biofilm formation, amoeba-associated lifestyle, and resistance to chlorine have been recognized as important factors that contribute to the survival, colonization, and persistence of MAC in water distribution systems [93, 94].

## 8. Diagnostic Techniques

The diagnosis of MAC is based on clinical signs, postmortem gross lesions in animals, culture, and also acid-fast staining or biopsy samples of fluids or organs.

### 8.1. Microbiology-Based Methods

The basic method for detection of mycobacteria remains cultivation using solid and liquid media [95].* MAC* grows best in egg based media and special agar media. The optimal incubation temperature for MAC species is 37°C–40°C. Culture can be performed in Dorset's and Herrold's egg yolk medium, Middlebrook 7H10 and 7H11 agar [20, 96]. Cultures should be incubated for at least 8 weeks. Nonsterile specimens need to be processed with detergent, alkali, or acid to eliminate rapidly-growing microorganisms. Incubation with various decontamination agents such as 0.6–0.75% hexadecylpyridinium chloride (HPC) or NaOH has been practiced. It is important that decontamination does not remove too many viable* Mycobacterium* cells [97]. Shorter incubation times can be achieved using automated broth based systems, like the liquid culture BACTEC system MGIT 960 [98]. These systems have been reported to be highly sensitive for culture [98].

Identification of mycobacterial isolates can be based on growth rates, colony pigmentation, and biochemical tests such as niacin production, nitrate reduction, tween 80 hydrolysis, and growth on MacConkey or sodium chloride tolerance [62]. The use of this approach for MAC members is limited due to the variable results for the majority of the applied tests. Conventional biochemical tests for species identification are time-consuming and fail to distinguish between* M. avium *and* M. intracellulare *[96].

Matrix-assisted laser desorption ionization-time of flight mass spectrometry (MALDI-TOF MS) is a rapid and highly promising tool for rapid primary differentiation of mycobacteria. However, routine identification is still hampered by the lack of procedure standardization and the small size of databases, even though some progress has been made in the development of new protocols. The accuracy of typing MAC subspecies by MADI-TOF can also be influenced by small differences in ribosomal protein structure [99].

### 8.2. Molecular-Based Methods

The development of molecular-based methods has enabled the differentiation of members within the MAC.

#### 8.2.1. Detection Kits Based on Hybridization Probes

Several commercial methods for the detection of mycobacteria using hybridization probes are available, including the AccuProbe system (Genprobe, San Diego, Calif), INNO-LiPA (Innogenetics, Ghent, Belgium), or GenoType* Mycobacterium* assay (Hain, Lifescience, Germany). Such tests can provide the desirable sensitivity and specificity but are very often limited to the identification of a small number of mycobacteria [100].

#### 8.2.2. Mycobacterial Broad-Range PCR Detection and Subsequent Sequence Analysis

Most mycobacterial species still lack known specific DNA loci suitable for PCR detection. Generally, broad-range PCR detection is targeted at common shared bacterial genes (via genus-specific primers), and subsequently, direct sequencing (Table 4) and sequence homology analysis are used to detect and differentiate mycobacteria [101]. Amplification of the gene to be sequenced preferably uses DNA extracted from a pure bacterial culture but can be achieved also directly from a clinical sample [102].

The first choice is generally the* 16S rRNA* gene [12], but the use of this target does not allow the discrimination of different MAC members. Other more variable regions are preferably used as second-step sequencing targets: for example, the internal transcribed spacer (ITS), for the differentiation of the members of the* Mycobacterium avium-intracellulare* complex, and the* rpoB*, for the rapidly-growing mycobacteria [19, 103].

#### 8.2.3. Species-Specific PCR Detection of Mycobacteria

Some MAC species can be differentiated based on the detection of insertion sequences (IS). The genome of MAA harbors the specific insertion sequence IS*901* and a low number of copies of IS*1245* [85, 104]. In contrast, MAH lacks IS*901* and usually contains a high number of IS*1245* [19]. The genome of MI does not contain the aforementioned IS elements [105].

#### 8.2.4. Genotyping Methods of* M. avium*

Molecular epidemiology is one of the areas in mycobacterial research which is widely used to study the transmission epidemics and outbreaks. It exploits the presence of various polymorphisms in the genome of the bacteria that can be widely used as genetic markers. Molecular-based techniques used in epidemiological studies to identify the source and origin of MAC infection are very well established in veterinary medicine. In human patients, however, such studies are performed only occasionally. Isolates can be typed using analysis of the restriction fragment length polymorphism (RFLP) pattern in the amplified IS*1245*, IS*1311* (MAH; [106]), or IS*901* (MAA, [107]). Another option is the typing of variable-number tandem repeats (VNTRs) of genetic elements called mycobacterial interspersed repetitive units (MIRUs) using PCR analysis [108].

#### 8.2.5. Next Generation Sequencing Technologies

A new prospect towards molecular epidemiology was introduced with the development of the next generation sequencing (NGS) methods, where the entire genome is sequenced which not only helps in pointing out minute differences between the various sequences but also saves time and the cost. NGS is also found to be useful in metagenomics studies, comparative genomics, and also various aspects about transmission dynamics. These techniques enable the identification of mycobacterial strains and also facilitate the study of their phylogenetic and evolutionary traits. Various platforms of NGS are being used widely (e.g., Illumina/Solexa Genome Analyzer; Applied Biosystems Ion Torrent; Helicos Heliscope*™*; Pacific Biosciences Single Molecule Real Time, SMRT).

As whole genome sequencing (WGS) becomes popular and implemented to research laboratories, new data with mycobacterial genomes were released and published [109, 110]. The comparison of whole genomic sequences obtained for relatively very close mycobacterial species would bring additional data useful for improvement of molecular-based bacterial typing. WGS is considered as an important tool to determine sequence variation at a real epidemiological scale, to determine the evolutionary relationship of the strains and also to determine the source of infection and the transmission of the disease between various patients [111]. WGS may become the gold standard for typing various strains for ME but there are certain limitations such as the need for specialized software to analyze the various sequence reads produced and the incomplete understanding of the genome variance.

## 9. Conclusions

The MAC comprises slow-growing mycobacteria that are ubiquitous in the environment, have a wide source range, and cause disease in a variety of domestic or wild animals and humans. The epidemiology of MAC infections is unclear in many cases, although colonization of the environment (mainly by MAH and MI) is well known and the prevalence of the disease in humans is increasing. Routes of transmission have not been clearly identified and transmission from human to human has yet to be convincingly demonstrated. The environment is the main source of MAC infections in humans.

Evidence for zoonotic potential should not be neglected, particularly with regard to immunocompromised patients. Recent reports, suggesting an association between MAC and autoimmune and other chronic human diseases, underline the importance of further research on MAC biology, diagnosis, and epidemiology.

Finally, it is critical to understand the impact of MAC in public health and to determine transmission routes between humans and wildlife. Such studies will require interdisciplinary collaboration among veterinary, medical, and other public health officials.

## Figures and Tables

**Table 1:** *M. tuberculosis* and *M. avium* complex taxonomy.

Member	Etiology
*M. tuberculosis* complex	
*M. tuberculosis*	Human tuberculosis, occasionally in animals in close contact with infected patients
*M. bovis*	Tuberculosis in bovines and other animals; humans are mainly infected by drinking raw milk
*M. bovis *BCG	The vaccine strain
*M. caprae*	Tuberculosis in bovines and other animals; humans are infected through the same pathways as for *M. bovis*
*M. microti*	Tuberculosis in rodents, rarely other animals and humans
*M. canettii*	Human infection, not detected in animals
*M. africanum*	Human tuberculosis, rarely in animals
*M. pinnipedii*	Tuberculosis in pinnipeds; animals bred in captivity are the main source of infection for caregivers

*M. avium* complex	
*M. avium *subsp. *avium* (serotypes 1–3)	Avian tuberculosis, mycobacterioses in pigs and humans
*M. avium *subsp. *hominissuis *(serotypes 4–6, 8–11, and 21)	Pig tuberculosis, human mycobacterioses
*M. intracellulare *(serotypes 7, 12–20, and 22–28)	Human infections
*M. colombiense*	Human infections
*M. chimaera*	Human and animal infections
*M. arosiense*	Human infection
*M. marseillense*	Human infection
*M. vulneris*	Human infection
*M. timonense*	Human infection
*M. bouchedurhonense*	?

?: yet to be confirmed.

**Table:** (a) Pulmonary localization

Signs		Laboratory findings		Signs
Bronchiectasis	++	Repeated massive isolation	+++	Cavitary, HIV
Nonspecific, CF, and COPD^†^	+/−^*∗*^	Repeated isolation	+++	Systemic
	−	Single isolation	+^*∗*^	
Low benefit of antibiotic treatment versus adverse effects Consider long-term observation Supportive therapy			Antibiotic treatment necessary	
Colonization: COPD, CF, coniosis-mild course, mycobacterial overgrowth-exacerbation; infection: chronic course, CT (RTG) progressive changes, and systemic signs.				

**Table:** (b) Extrapulmonary localization

Signs		Laboratory findings		Signs
Skin, soft tissue	+++	Repeated massive isolation	+++	HIV, systemic organs
Lymphadenitis	++	Repeated isolation	++	Osteoarticular, tenosynovial
	+/−^*∗*^	Single isolation	+	
AT therapy necessary Lymphadenitis in children, skin: extirpation of affected lymph nodes is of high importance, excision of affected tissue; osteoarticular, tenosynovial, chirurgical debridement; HIV, Haemocultivation.				

^+++^Strong recommendation for treatment.

^++^Recommendation for treatment.

^+^Weak recommendation for treatment (repeated sample collection necessary), managing intermittent treatment.

^−^No treatment.

^*∗*^Based on isolated MAC species. From invasively taken sample (biopsy, bronchoscopy).

^†^Consider contamination, repeated exposition to environmental source, and household screening.

**Table 3:** Antibiotic regimes, summarization.

	Combination	Duration	Comments
Pulmonary			
Cavitary disease Pneumonia	Clarithromycin 500–1000 mg/d or Azithromycin 250–300 mg/d Rifampicin 450–600 mg/d	Up to 12 months, negative culture Initial administration of injectable agents for 8 to 12 weeks Streptomycin (10–15 mg/kg/3x/week) Amikacin 8–25 mg/kg 2-3 times weekly	In case of macrolide resistance or intolerance clofazimine and quinolones may be useful based on strain resistance profiles
Bronchiectatic-fibronodular CF, COPD	Clarithromycin 1000 mg/d or Azithromycin 500–600 mg/d Ethambutol 25 mg/kg Rifampicin 600 mg	Intermittent three times weekly In nonresponsive output change to daily dosage	Adjuvant therapy of underlying disease, bronchodilators, airways clearance support, smoking cessation, and nutrition
Hypersensitivity	Prednisone 1-2 mg/kg/daily	4–8 weeks, repeated exposure must be avoided	Long postexposure observation to exclude lung disease progression

Extrapulmonary			
Systemic, disseminated, and HIV infection	Clarithromycin 500 mg/twice daily or Azithromycin 1200 mg/daily Rifabutin 300 mg/d Ethambutol 15 mg/kg daily	Up to 12 months Initial administration of injectable agents for 8 to 12 weeks Streptomycin (10–15 mg/kg/3x/week) Amikacin 8–25 mg/kg 2-3 times weekly	Prevention Azithromycin 500 mg/daily Ethambutol 15 mg/kg daily or Clarithromycin 500 mg/twice daily +/− Rifabutin 300 mg/daily, HIV with < 50 CD4 cells/L
Skin, soft tissue, and osteoarticular disease	Chirurgical debridement, excision Support: Clarithromycin 500–1000 mg/d or Azithromycin 250–300 mg/d Rifampicin 450–600 mg/d	In case of indolent persistence, supportive in more excessive involvement: Streptomycin (10–15 mg/kg/3x/week) Amikacin 8–25 mg/kg 2-3 times weekly	
Lymphadenitis	*Complete extirpation* Support: Clarithromycin Rifampicin	Duration and dosage not established,see pulmonary, consider Ethambutol with caution in infants!	Antibiotic treatment only in cases where extirpation is contraindicated or with poor outcome after surgery, considering superinfection with other microorganisms

**Table 4:** The most frequent sequencing targets used for identification of nontuberculous mycobacteria.

Target gene	Name	Primer type	Sequence 5′ → 3′	Length of the product (bp)	Reference
*16S rRNA*	16S27f 16S907r	Forward Reverse	AGAGTTTGATCMTGGCTCAG CCGTCAATTCMTTTRAGTTT	921	Harmsen et al., 2003 [12]

*hsp65*	Tb11 Tb12	Forward Reverse	ACCAACGATGGTGTGTCCAT CGGATCACTCGTGAACGCTA	441	Telenti et al., 1993 [13]

ITS	16S-1511f 23S-23r	Forward Reverse	AAGTCGTAACAAGGTARCCG TCGCCAAGGCATCCACC	Approx. 380^*∗*^	Harmsen et al., 2003 [12]

*rpoB*	Myco-F Myco-R	Forward Reverse	GGCAAGGTCACCCCGAAGGG AGCGGCTGCTGGGTGATCATC	764	Adékambi et al., 2003 [14]

^*∗*^Amplicon size is not uniform due to the 16S-23S spacer length variability.
